# PHARMACY, MONEY AND PUBLIC HEALTH IN DAKAR

**DOI:** 10.1017/S0001972013000454

**Published:** 2013-11

**Authors:** Noémi Tousignant

## Abstract

Pharmacy students at the Cheikh Anta Diop University in Dakar must research and write a thesis to graduate. *Thésards* who took topics in analytical chemistry and toxicology describe their thesis work as a temporary opportunity to perform ‘street-level’ public health research that they regard as ‘relevant’ to the quality of people's lives. Expecting futures in the private commercial sector, *thésards* regretfully leave the thesis behind. This article explores the parenthetical nature of this moment – its brief openings and more durable closures – as part of the history of ways of being a pharmacist in post-colonial Senegal. The thesis as an interlude in students’ biographies, curtailed by narrowed horizons of expectation, evokes other contractions: in the range of professional roles open to Senegalese pharmacists, and in the circuits of public health with which they might engage. For *thésards,* fieldwork, government work and commercial work entail spatial practices and imaginations; different ways of moving around the city and of tracing urban space that define pharmacists’ roles in terms of the modes through which they engage with broader collectivities. Mapping *thésards’* parenthesis in Dakar is a means of capturing both their urban experience of work and the intertwining spatial, temporal and affective dimensions associated with this work. The past, probable and possible trajectories of pharmacy work are imprinted and imagined in the space of the city as field, market and polis. Pharmacists’ prospects and aspirations are caught up in broader shifts in how education, (un)employment and entrepreneurship animate relations of association and exchange in Senegal.

## RÉSUMÉ

Afin d'obtenir leur diplôme, les étudiants en pharmacie à l'Université Cheikh Anta Diop de Dakar doivent préparer et rédiger une thèse. Parmi les thésards qui ont pris des sujets de thèse en chimique analytique et toxicologie, certain(e)s décrivent ce travail comme opportunité temporaire de faire de la recherche en santé publique “au niveau de la rue,” c'est à dire pertinente au bien-être des populations. Anticipant un avenir dans le secteur commercial privé, les thésards regrettent devoir tourner la page sur ce travail au terme de leurs études. Cet article explore la nature parenthétique de la thèse, en tant que moment de brèves ouvertures qui se referment ensuite durablement, et la situe dans l'histoire des manières d’être pharmacien au Sénégal post-colonial. La thèse apparait comme interlude dans la biographie des étudiants, écourtée par le resserement de leurs perspectives d'avenir, evoquant ainsi d'autres contractions: dans l’éventail des rôles professionnels et dans les circuits de santé publiques ouverts aux pharmacien(ne)s sénégalais(es). Le travail de terrain, le travail fonctionnaire et le travail commercial comportent pour les thésards des pratiques et imaginations spatiales ; différentes manières de se déplacer dans la ville et de tracer l'espace urbain, chacune définissant le rôle pharmacien en terme de ses modes d'engagement avec les collectivités. La projection de cette parenthèse sur la carte de Dakar nous donne un moyen de saisir à la fois l'experience urbaine de leur travail de terrain et l'imbrication de ses dimensions spatiales, temporelles et affectives. Les trajectoires passées, probables et possibles du travail pharmacien s'imprègnent et sont imaginées dans l'espace de la ville en tant que terrain, marché et polis. Les perspectives et aspirations des pharmacien(ne)s relèvent de dynamiques historiques plus larges, changeant la manière dont l’éducation, l'emploi (et le chômage) et l'entrepreunariat animent les relations d'association et d’échange au Sénégal.

‘I still think about pesticides!’ Madior^1^ has taken time from his work in the local production unit of a multinational pharmaceutical firm to tell me about the passion he developed for the topic of his pharmacy thesis a few years earlier. This thesis is a requirement for graduation from the pharmacy programme at Cheikh Anta Diop University in Dakar. Madior studied the impact of agricultural practices on levels of pesticide residues in vegetables in the Dakar market. Despite his enviable job, Madior complains that pharmacy graduates ‘are forced to go towards the private sector’. Few work in firms, as Madior does. Some obtain coveted positions as pharmaceutical company marketing representatives. The majority end up, usually after a period of unemployment or assistantship, running a private pharmacy. But ‘That's not the goal!’ Aisha exclaims. Her thesis surveyed dentists’ knowledge of pharmaceutical side-effects. ‘I don't want to be just a [private] pharmacist,’ she says, ‘it's doing research that I'm interested in. I love public health. I prefer to move … just be useful, I mean … for the Senegalese population.’ Fatma, who did her thesis research on pharmacists’ knowledge of intoxication, dreams that ‘given the means, I would take any topic, I would go to the end of the world [for research]’.

Over the course of five years, pharmacy students are taught much more than how to handle medicines. In lectures, laboratory exercises and internships in private pharmacies and public hospitals, they learn skills suited to jobs ranging from running a biology lab to analysing medicinal plants or monitoring water quality. In their sixth and final year, they research and write an original thesis. Many theses entail fieldwork and most claim to contribute, even if modestly or indirectly, to public health and safety. When I spoke to thesis-year students or recent graduates who had taken topics in toxicology or analytical chemistry, they evoked this work as a fleeting opportunity to formulate and pursue goals that were at once civic and scientific. In fieldwork, they discovered how research could illuminate specific public health risks at ‘street level’: in contaminated foods and substandard medicines that circulate in markets; in unsafe or uninformed practices located in fields, homes or beauty salons across and around the city. But it soon came to seem unlikely, for most, that they would ever practise science as a public service again. The time of the thesis was cut off by their awaited or achieved entry into industry, marketing and commerce. *Thésards* thus evoked the thesis not as a transitional threshold but as a parenthesis in their passage from student to professional; a moment suspended between many possible ways of being a pharmacist and the limited range of opportunities offered by the Senegalese job market.

This article explores the parenthetical nature of this moment – its brief openings and more durable closures – as part of the history of ways of being a pharmacist in post-colonial Senegal. What does thesis work open onto? What kinds of work do *thésards* evoke when they speak of passion for pesticides, fieldwork and public health? How do they contrast thesis work with their probable futures, and what do they stand to gain and to lose in the transition? Through such questions, the thesis as an interlude in students’ biographies, curtailed by narrowed horizons of expectation, evokes other contractions: in the range of professional roles open to Senegalese pharmacists, and in the circuits of public health in which they might engage. By relegating science, research and public health to a parenthetical moment, *thésards* point to ways in which historical trends (in public regulation and employment, the market for pharmaceuticals and the field of pharmacy) have shaped their career possibilities. They also express affective positions – of pride and regret, ambivalence and hope – with respect to past, probable and possible ways of being a pharmacist. Do they expect and aspire to making profits or knowledge, running government services or private businesses, engaging with publics or markets? By situating ‘street-level’ thesis work in terms of broader patterns in public health and in pharmacists’ employment, I take the parenthesis – the temporal and affective qualities of thesis work evoked by *thésards* – as a starting point to explore how, over time, tensions as well as juxtapositions emerge among pharmacists’ roles in this particular setting.

By separating thesis research and private employment in their subsequent careers, *thésards* cast as opposites two types of roles for pharmacists: public/scientific on the one hand, and private/commercial on the other. This opposition is not a given; indeed, modern pharmacists have successfully staked claims to privileges – and associated civic obligations – on the basis of their dual vocation as businesspersons and health professionals (Faure 1996). In Senegal this duality has been upheld in historically specific ways; in the 1970s, subsidies to commercial pharmaceutical production and distribution were granted, while recently private pharmacists have claimed the right to a legally protected market by affirming their civic duty to act as guardians of public health (Tousignant 2013a). *Thésards’* separation of commercial from civic/scientific roles appears not as the statement of a fixed reality, but as a commentary on the opportunity, uncertainty and difficulty they face at a particular moment in their careers and in the history of Senegalese pharmacy.

The versatility of pharmacy – as a profession that can be commercial, regulatory, managerial, scientific or medical – thus takes historically specific forms. Observing a moment in young professionals’ lives when this versatility has not yet resolved might shed new light on the effects of structural adjustment and privatization not only on work opportunities, but also on the associated identities and subjectivities that arise from their pursuit. Rising unemployment and diminishing state capacity to provide access to employment, especially to university graduates, have certainly changed expectations about the channels, qualities and commitments needed to secure livelihoods and status (Boquier 1996; Leimdorfer and Marie 1998; Ralph 2008; Fall 2010). But does this necessarily mean embracing the spirit of self-reliance and entrepreneurialism? Pharmacy students’ ‘unresolved versatility’ opens to consideration how an affirmation of entrepreneurialism continues to coexist and sometimes clash with other forms of professional expertise and ethos.

## SENEGALESE PHARMACISTS AS FIGURES OF SUCCESS

The changing political and moral economies of African cities have led to the emergence of new modes of acquiring power, status and wealth. The introduction to a special issue of *Politique africaine* on ‘Figures of success and political imaginaries’ opens by stating that the figure of the functionary – salaried, state-employed and formally educated – has lost its value as an image of modern, urbane, social and material achievement (Banégas and Warnier 2001; see also Nyamnjoh 2005). It goes on to describe new modes of accumulation and styles of being that seem better adapted to conditions of rising unemployment and informal arrangements, of deterioration and privatization of public services and education, and of reconfigured, accelerated transnational circulations of goods, people and values. New itineraries of accumulation, the editors of this issue point out, are not simply ‘constructed … outside the state’ but engage with its new modes of intervention and lines of fracture (rather than its ‘retreat’) (Banégas and Warnier 2001: 7; see also Hibou 1999). This leads to a ‘refashioning of political subjectivities as well as a redistribution of moral reference points’, with the affirmation of an entrepreneurial ethos of cunning, risk taking and ‘survival of the fittest’ in strategies of both ascendance and survival (Banégas and Warnier 2001: 8; see also Petit and Mutambwa 2005).

The activation of such an opportunistic ethos is associated with new modes of entering and inhabiting the city. In Senegal, a prominent new figure of success is that of the *moodu moodu*, an urban merchant who now asserts his previously stigmatized rural origins, moves easily in transnational commercial and financial networks, and operates in the ‘interstices of … illegality’ (Banégas and Warnier 2001: 8). Senegalese urban youth movements – *set setal* (clean, clean-up) in the late 1980s and *bul faale* (don't bother) from the 1990s – have adopted local rappers, sports stars and religious figures as icons. Mobilized by youth to represent new values of self-reliance and individual effort in critique of the corruption, hypocrisy and irresponsibility they associate with the older generation of post-colonial politics and nationalism, these figures – represented in murals, graffiti, street and commercial names – have displaced older national and African heroes in Dakar's geography (Diouf 1996; Havard 2001). Transnational migrants render their earnings visible and stake their claims in Dakar by building houses, distinctive in their size, style and gradual completion (Tall 2009; Melly 2010). Other young Dakaroises are ‘ready to sacrifice anything, including morality’ to gain access to the pervasive but exclusionary consumer culture commanded by these new figures of success, such as the star or the migrant; turning themselves into figures of cunning, trendiness and seduction (Nyamnjoh 2005). The urban mobility and material visibility of the *moodu moodu*, the *bul faale* icon, the migrant or the modern seductress (*disquette*) stand in contrast with the circuits of instruction, service, civility and reward travelled by the functionary in the post-independence decades. This route led from the university, often via advanced studies in France, to modernist administrative buildings and public institutions, and back home, riding in cars or buses to houses that were state-subsidized from the 1950s onwards, largely for the benefit of this category of earners (White 1985; Freund 2006; Lombard and Zouhoula Bi 2008).

Against this backdrop of obsolete and novel ways of ‘making it’ in Senegal, the pharmacist appears as a remarkably durable figure of success with a stable urban presence. Rising enrolment figures at the Faculty of Pharmacy in Dakar – the total number of students grew from 742 to 1,212 between 1983 and 1987, remaining stable at around 1,000 until at least the mid-1990s (Gaye 1995) – seem to indicate the continued popularity of this course of study as a road to opportunity. Old and new private pharmacies mark an aesthetic of prosperity at regular intervals across the Senegalese landscape. The number of pharmacies nearly doubled in the 1990s, and again in the 2000s to reach 870 in 2009, largely concentrated in urban centres and especially Dakar. Urban pharmacies with middle-class clienteles are luxurious, offering imported cosmetics as well as baby products and nutritional supplements, but even more modest businesses in Dakar's so-called popular neighbourhoods stand out with their distinctive façades of green and white patterns, selling price-controlled generic drugs prescribed by public dispensaries, or without a prescription, saving their poorer clients the cost of a medical consultation. Growth in the number of graduates and businesses seems to show how well the figure of the pharmacist has aged; already considered to be a ‘smart’ career choice in the 1970s, it is still seen as a lucrative and respectable option for both men and women (Barthel 1975).

Pharmacists, like other figures of success who came after them, appear to have been agile in entering and occupying new niches of opportunity (Banégas and Warnier 2001). Tightly entwined with an expanding state through subsidies and employment in the post-independence decades, Senegalese pharmacists once embodied figures of success associated with responsibility for public health and national development (Tousignant 2013a). With the expansion of the private sector and the stagnation of government structures, hundreds of graduates have used their qualifications to sell medicines. At a time when a degree no longer guarantees employment, they, like other graduates, have had to deploy entrepreneurial skills to make a living (Boquier 1996). Many will never run their own pharmacies, and many pharmacies have trouble making ends meet; and yet the pharmacist as an independent businessperson or a slick ‘rep’ is seen as a figure of success in post-adjustment Senegal, a ‘winner’ in the reorientation of opportunities and obligations.

When graduating students speak about their thesis work and career opportunities, what do they say about the outdating and emergence of styles of success in Senegal? Do they feel that an entrepreneurial ethos has displaced older or alternative ways of being good or successful through pharmacy? Before providing a more detailed history of Senegalese pharmacy careers, I say a bit more about how my encounters with *thésards* generated such questions about loss and opportunity in the public protection and marketplace of health.

## PLACING A PARENTHESIS

Over the course of two weeks in February 2011, and again during brief follow-up research in February 2013, I spoke to 14 imminent or recent pharmacy graduates about their thesis work.^2^ As part of a pilot project on street-level health work, my initial interest was in the urban fieldwork they had conducted for their theses. Most seemed puzzled by this interest. They gave rehearsed descriptions of the topic itself, probably echoing the ‘aims and objectives’ they had written up, or, for those who had graduated, presented in their public defence of the thesis. They were less eloquent about the details of fieldwork, unsure why I would want to hear about the elaboration and correction of questionnaires, the laborious journeys by foot and bus across the city, the people they interviewed and the neighbourhoods they visited. All became noticeably more animated, however, when they explained why their topic was significant, particularly in terms of public health.

Supervisors generally ‘hand out’ thesis topics on the basis of their own judgement of significance and feasibility. A few *thésards* claim they chose a topic, or immediately grasped its value, because they ‘know’, and care, about a specific problem: thus Fatma, for example, was concerned about pharmacists’ ability to be frontline respondents in the management of acute intoxications. For most, says Mamadou Fall, a professor of toxicology, ‘at the beginning, it's just to get their diploma … but eventually … they *see*’. Angélina, to whom Fall had allocated an investigation of household pharmacies, corroborated this outcome: ‘When Fall gave me the topic, I just wanted to do it, analyse, finish. … But then I invested myself in the work. I understood [that the way people handle medicines at home] was dangerous.’ Oriented towards pharmacy studies by her father and a private pharmacist uncle as a course with good prospects, Angélina was initially ‘not very motivated’ but held on. She, not her parents, insisted she re-try after a failed year, and she became ‘passionate about nutrition, quality control, public health’. Angélina would have preferred to work on pesticide residues or water quality. But when she set out to do her literature review on household pharmacies, she found no studies conducted in francophone Africa. Mariama encountered another kind of information gap when she set out to survey hairdressing salons, which, not being subject to regulation, were not registered and had to be located ‘on the street’. Through such banal details emerges a sense of entering terrain unexplored by inquiry and regulation. *Thésards’* animation about their contribution thus seems to manifest pride in the achievement of something difficult (sighs and exclamations of ‘it was hard!’ were recurrent), something new (for Senegal or West Africa), and something worthwhile (‘for the population’).

Although admittedly the *thésards* I spoke to were a small sample, they expressed remarkably similar pride in their thesis work, which then segued – spontaneously for some, for others in response to my questions – into ambivalent, resentful or even defiant attitudes towards current or future employment in commercial work. This surprised me as an attitude towards what is, by most accounts, a very tight job market. Students and faculty frequently comment on the inability of this market, commercial or otherwise, to absorb the large number of pharmacy graduates and the periods of unemployment many consequently face. Data on the high proportion of retail pharmacies that are ‘facing difficulties’ (20–25 per cent) can be found in newspaper articles and a pharmacy thesis (Diaw 2004; Sonko 2010). Yet the students I spoke to identified private pharmacy ownership as a likely and lucrative destiny. Some seemed to accept this, but still wished they had other options. Others were more determined to find a way into research or public health, though they were far from confident this was possible, and saw commercial work as a default or a ‘last resort’. All spoke in some way of being ‘forced into the private sector’, whether, like Madior, they were already privately employed or, like Aisha and Fatma, were still unsure about their prospects.

This ambivalence towards commercial work is perhaps a response to uncertainty, the expression of desire for greater opportunities. Yet it is not just a call for more job openings, but also for a greater range of professional roles, including ones that echo thesis work, carrying the promise of ‘doing something for the population’ through scientific testing or research. A few students, such as Fatma and Leonie, spoke of the (potential) public health role of private pharmacists – in ensuring the accessibility of medicines; providing frontline healthcare; or protecting therapeutic safety. Indeed, Fatma and others have made the improvement of these capacities the topics of their theses. Senegalese private pharmacists have conducted a well-publicized campaign for the protection of their professional privileges (by cracking down on illicit pharmaceutical sales) and their inclusion in well-funded treatment programmes. These demands on the state are supported by claims that they are, in their ethos and expertise, ‘private agents of public health’; in other words, more than ‘just merchants’ (Tousignant 2013a). Yet the *thésards* I spoke to clearly position thesis work in opposition to private pharmacy. Why? Are they simply anticipating, soon after the excitement and discovery of thesis as *research*, the dullness of dispensing medicines? Are they just anxious about their prospects and longing for the security of the now non-existent civil service jobs that thesis work – with its orientation towards public monitoring and regulation – evokes?

Expressions of attachment to public health and of ambivalence about ‘just’ making money may be momentary, emerging briefly between the pride of completing a degree and an uncertain future. They may be circumstantial to conversations about the significance of thesis topics. Are these expressions consistent and durable? It would certainly be interesting to explore the range of ways in which pharmacy students aspire to both service and success; how, throughout their training, they motivate themselves to pass academic hurdles, develop ‘passions’ through internships, sign up for medical outreach in student-organized ‘civic holidays’, or admire the car of a fellow student who works part-time as an industry ‘rep’. One might also ask older private pharmacists about their past dreams and ongoing commitments. This article does not place the parenthesis evoked by *thésards* among pharmacists’ multiple aspirations – to mobility and success, profit and service, knowledge and efficiency – as they unfold over time, whether over the course of their training or their careers (on the value of ethnographic study of medical training to explore issues of civic commitment in Africa, see Wendland 2010). Instead, I focus on the specificity of this moment; a moment in which young pharmacists poised on the cusp of their careers contemplate the traces of multiple past trajectories – of an older generation of pharmacists coming to the end of careers of public service, of the hundreds of graduates before them who have dotted Senegal's streets with private pharmacies, of their own field circuits as *thésards* – and the futures these have made probable as well as imaginable.

I thus place this parenthesis within Senegalese pharmacists’ broader career patterns, drawing mainly on documentary research, but also on oral histories, and an historical analysis of pharmacists’ positions and circulations between private and public spheres (Tousignant 2013). I also place this parenthesis in terms of what it contains – its objectives, methods, conclusions and recommendations – with respect to the specific public health actions and issues with which this work engages. I focus on *thésards* (14 interviewees) and theses (217 reports) supervised by members of the laboratories of toxicology and of analytical chemistry (formerly a single administrative unit, recently split into two) in the Faculty of Pharmacy of Cheikh Anta Diop University in Dakar. This forms a manageable sample of students and theses, and circumscribes a set of topics addressing the public health issues associated with these sciences (food, drug and chemical safety). Lab members, who are functionaries appointed as faculty of this public university but also often by the Ministry of Health in its various services, have a longstanding engagement with these topics through research (namely the research undertaken by ‘their’ *thésards*) but also in key Ministry of Health institutions such as the Laboratoire National de Contrôle des Médicaments (LNCM, National Drug Control Laboratory) and the recently created Centre anti-poison (CAP, National Poison Control Centre). I interviewed them about thesis supervision but also more broadly about the regulation of food and drug quality and I was a participant-observer at the CAP for several months in 2010. This allowed me to situate thesis work with respect to existing efforts to regulate the public health risks posed by toxins, contaminants and fraud.

Both pharmacists’ career patterns and circuits of public health, I suggest, are best understood when located in Dakar. I first approached thesis work as ‘urban’ because, since the 1990s, a growing proportion of toxicology and analytical chemistry theses entail data collection, and in the majority of these (125 out of 156) data are obtained from sampling in Dakar. When, on rare occasions, *thésards* have had access to working lab equipment at the university or elsewhere, they have brought in tea, drugs or air samples from Dakar's streets to analyse for conformity and contaminants. More often, they have set out with stacks of questionnaires or forms to record responses, data or observations. Many *thésards* thus experience thesis work as moving and locating sampling units across Dakar. Except in rare cases, this does not identify their research topics as pertaining to *urban* health. Dakar is usually chosen as a field for practical reasons, because students cannot or will not pay to go elsewhere unless they are going back home, and because the city's density allows for easier sampling. At the same time this density allows for trajectories across heterogeneous terrain, with sampling sites often chosen to reflect socio-economic diversity, which, depending on the topic, allows students’ sampling areas to represent the effects of what they sample (potentially contaminated matter, or of professional and artisanal practices) on a broader population.

Dakar is also where young pharmacists study and are most likely to work. In late 2009, 506 of Senegal's approximately 870 pharmacies were located in Dakar, making recent growth in private pharmacies most visible here. While the retail market for pharmaceuticals appears to be nearing saturation, it still continues to expand with Dakar's demographic growth, which is fuelled by the migration of rural residents seeking livelihoods, but also exhibits increasing signs of middle-class consumption (strikingly visible, over the past few years, in sleek new apartment buildings, dense road traffic, burgeoning supermarkets and shopping centres, and billboards advertising luxury products). Wholesale firms, pharmaceutical manufacturers and the main government institutions employing pharmacists are also concentrated in the greater Dakar area. There has been some growth in private firms, particularly those seeking to distribute generic drugs in Senegal, but the number of public institutions and employees has remained fairly stable while the number of pharmacy students has grown (Gaye 1995). Thus the young pharmacists’ view of the job market is superimposed on their sense of Dakar as a growing but highly competitive private market for pharmaceuticals. Yet most are also aware that this was not always the case: they associate career trajectories through public institutions of education and health with the expectations and experiences of a prior generation.

Listening to *thésards,* my interest shifted from the ways in which their field itineraries rendered the city legible as a space of toxic risk to how they set fieldwork in contrast with government and commercial work. These different ways of being a pharmacist are neither neutral nor fixed; they have affective qualities associated with, for example, duty and success, and they are contingent on specific historical conditions. They also entail spatial practices and imaginations; different ways of moving around the city and of tracing urban space as linked or fragmented, exclusive or encompassing, saturated and bounded, or open and expansive. But more importantly for pharmacists’ experience of the city as a field and workplace, these movements define their roles in terms of the modes – commercial, scientific or governmental – through which they engage with broader collectives. Placing *thésards’* parenthesis in Dakar is a means of capturing both their urban experience of work and the intertwining spatial, temporal and affective dimensions associated with this work. The past, probable and possible trajectories of pharmacy work are imprinted and imagined in the space of the city as field, market and polis. This leads not only to the city as a changing institutional, demographic and economic (as well as epidemiological) landscape that shapes the need and demand for pharmacists’ work. It also opens onto the city as a site in which they position themselves as experts and elites in relations of service and obligation, profit and privilege, with respect to the state and ‘society’. Pharmacists’ prospects and aspirations are caught up in broader shifts in how education, (un)employment and entrepreneurship animate relations of association and exchange in Senegal (Boquier 1996; Leimdorfer and Marie 1998; Ralph 2008).

## WORKING IN THE CITY

Mounirou Ciss became the first Senegalese professor of toxicology not by following his ‘childhood dream’, he told me, but by choosing the science where ‘there was no one’ (meaning no Senegalese student set to replace the current French technical assistant). Private pharmacy did not attract him because it forced one to choose ‘between money and family’, that is, between focusing on profit or redistributing medicines and gains within solidarity networks. Instead, he could escape this dilemma by relying on his talent for science to pursue his studies, accede to a rapidly tenured position at the university, then head one of the main public hospital pharmacies at a time when it was a nodal point in Senegal's pioneering public anti-retroviral access initiative in the late 1990s, and, finally, become a spokesperson for the revitalization of drug quality control as director of the rising LNCM in the 2000s. While initially he may not have aspired to it, his career stands as an exemplar of commitment to science, the state and public health.

Ciss's counterpart in analytical chemistry, Doudou Ba, had a similar trajectory through high positions of public authority, and has retired to become permanent secretary of the National Academy of Science and Technology. Ba tells me that two of his contemporaries, a small cohort who obtained public scholarships for advanced studies in France and returned in the mid-1970s, tried and failed to run private pharmacies, but were reintegrated in the public system, ‘because we couldn't lose people that Senegal had paid to educate’.^3^ All became prominent public figures whose political and scientific achievements are widely known: Issa Lo and Mamadou Badiane have been directors of the pharmacy section of the Ministry of Health, and known to be active in the development of Senegalese and African pharmaceutical regulation, while Balla Moussa Daffé has been delegate to the National Assembly, mayor, regional counsellor and Minister of Scientific Research and Technology. Other well-known figures are the slightly older Mahjemout Diop, who is said to have used the profits from his private pharmacy, enabled by public scholarships and loans, to finance a career as a radical politician, and the slightly younger Souleymane Mboup, who has become a national and African hero for the co-discovery of the HIV-2 virus.^4^

This first generation of Senegalese pharmacists entered a city of expanding government. The 1960s and 1970s are more generally remembered as ‘the glorious era of full employment guaranteed by the State to university graduates’ (Fall 2010: 311). The urban fabric bears the marks – state buildings, subsidized housing and transportation, civil servant salaries – of the relationship between government and the educated elite, animated by a discourse of shared commitment to national development (Tall 2009). For the relatively few pharmacists graduating from the Faculty of Pharmacy in Dakar, this translated into government bursaries, employment, or low-interest loans meant to Senegalize pharmacy education, regulation and business. The city filled with visible signs of this policy. A new building for the Faculty of Pharmacy was inaugurated in 1966 to turn out pharmacists needed to fill public positions (Attisso 1970). Several students were sent for advanced training in France during the 1970s. Returning to replace French technical assistants as lecturers, they were immediately cross-appointed to positions at the Ministry of Health. They became pharmacy inspectors, members of new expert committees advising on education and importation, or chiefs of hospital and national pharmacies and laboratories.^5^ Their trajectories across the city – between the university and public teaching hospitals or administrative units – linked pharmaceutical expertise to the state and public service.

Senegalization also stimulated a wave of growth in private pharmacy institutions. In the mid-1970s, the state began providing low-interest loans to help Senegalese pharmacists set up private retail and wholesale pharmacies (Ba 1977). These pharmacies would sell some medicines that were partly produced and distributed by the state. After several government-sponsored studies on publicly funded local pharmaceutical production, the state in 1973 signed an agreement with a European pharmaceutical firm to create the SIPOA, a mixed public-private production facility that was set up in Dakar's industrial zone (SEDE 1969; SONEPI 1970; SOFFIN n.d. [*c*. 1972]). Public spending (on salaries and subsidies, or, indirectly, through tax cuts) thus entangled professional mobility with an urban presence – in institutions and business – and circuits (of sale and of double-appointments) for young Senegalese pharmacists. For a brief time during the 1970s, these movements across the city drew pharmacists into a relation with the state of shared responsibility for national construction.

Cuts in public budgets would soon change how pharmacists might travel from the university into the city. Most obviously, it has changed graduates’ expectations and strategies for future employment. While the number of students enrolled in the Faculty of Pharmacy nearly doubled in the mid-1980s, the state stopped hiring pharmacists with the implementation of structural adjustment measures (Knowles *et al*. 1994; Gaye 1995). Shortages of medicines in the public sector accelerated the growth in private pharmaceutical trading, both legal and informal (Fassin 1985, 1986; Brudon *et al.* 1997). Between 1985 and 2001 the number of private pharmacies increased five-fold, from 109 to 532 (Diaw 2004); by the end of 2009 there were estimated to be 870 (Sonko 2010).

The work patterns of those already in public academic and ministerial positions also changed. Lecturers seeking to supplement department budgets and salaries affected by reductions in public funding from the 1980s, and especially by currency devaluation in 1994, increasingly sought private contracts for laboratory analysis and teaching.^6^ Others, especially those in junior positions, left the university to work in the private sector. The implementation of the Bamako Initiative in the 1990s, advocating the promotion of generic drugs and cost recovery, reformed public structures to emulate private business models (Brudon *et al*. 1997; Fall *et al*. 2004; Foley 2010). Since about 2000, some public pharmaceutical institutions have received new injections of resources, public hiring has resumed slowly, and salaries in the public sector have been increased significantly (République du Sénégal 2010).^7^ Yet entrepreneurial styles and strategies continue to permeate public pharmaceutical institutions such as the Faculty of Pharmacy and the National Supply Pharmacy.^8^

Despite reported ‘overcrowding’ of the retail pharmacy sector, *thésards* still describe pharmacy ownership as a means of ‘making money without tiring oneself out’, as Aisha puts it. But the *thésards* I interviewed saw it as their default option, with other private sector jobs – in industry, distribution and marketing – as slimmer but still realistic possibilities. The kinds of trajectories they associate with the ‘pioneer’ generation of pharmacists, which they hear about in graduation ceremony speeches paying tribute to the ‘godfather’ (and more rarely ‘godmother’) of the class or even in news media, seem closed off. In the post-colonial decades, talented pharmacy students could expect to take on roles as scientific experts in government service. Now, graduates who want to take a shot at rare jobs in government and/or science must complete additional Masters degrees in hospital pharmacy or biotoxicology, for example, or specializations in sciences such as biochemistry; this requires not only tuition fees, but also putting off remuneration and accepting that prospects will remain uncertain. Aisha cannot ‘lose time’ waiting for an opening in the public sector, while Fatma cannot afford the Masters fees: ‘I don't have the means for attaining my ambitions. I would even say that I'm stuck. That pushes a lot of people to do something out of necessity’ – which for her means trying to get work as a ‘rep’ to save the money to study. Pharmacy students appear to come from privileged backgrounds: they are well-dressed and often confident, and with the deterioration of the public education system, most would have had to go to good private schools to make it this far. Like Angélina, many *thésards* I interviewed reported scientists or pharmacists in the family. I do not have extensive data on the socio-economic background of pharmacy students, but the length of the programme (six years, and re-sitting some years is not infrequent) means that they have, at least to some extent, been able to rely on the support of their families while qualifying. Thus, by choosing to study pharmacy, they seek to maintain middle-class status. Going further – to seek a career in research and/or public health, thus combining science, service and government, has become a more exclusive and uncertain privilege.

This does not mean that young graduates mourn for their predecessors’ trajectories. But they certainly seem to yearn (perhaps only briefly) for something more than what they feel they can expect. Why does talking about thesis work evoke this yearning?

## SAMPLING PUBLIC HEALTH

*Thésards* present their theses as adventures in public health. To understand this, I suggest we consider what (and where) this work samples. *Thésards* go out to collect something to analyse: a careful selection of potentially contaminated matter, or of responses to questions about risky practices and protective knowledge. This act of sampling is central to fieldwork-based theses in toxicology and analytical chemistry, for *thésards* rarely improve or validate analytical methods.^9^ Both the originality and significance for public health of thesis work depend on sampling. Foods and medicines sampled at points of sale (in shops or street-stalls, for example) are meant to be representative of what Senegalese are actually exposed to, rather than being limited to the better-monitored (by both government and industry) traffic into and out of the country. In surveys of practices relative to toxic chemicals and accidents, supervisors ask *thésards* to visit several neighbourhoods – including older, more central and urbanized ones (such as Medina) and suburban neighbourhoods such as Pikine and Guediawaye, with ‘popular’ (poorer, denser) as well as middle-class areas – thus capturing a broad socio-economic demographic. One supervisor thinks of topics he hands out as ‘pilots’, in which a survey conducted for the first time in Dakar will prepare its extension to other areas of Senegal (though this has not yet happened). Although *thésards* certainly do not cross or cover the city as a whole, or draw consistent patterns that could easily be mapped, the choice and distribution of sampling areas are clearly meant to point towards broader socio-political spaces that are expansive but within national boundaries.

Sampling as an element of research method leads to sampling in a second sense: in many cases thesis work forms a brief instance of what operational public health action might be. *Thésards* sample things and responses in locations and people that could be, but are not, enmeshed in networks for monitoring food or drug safety, or for diffusing information about safe ways of handing chemicals. In other words, it is useful for *thésards* to test vegetables sold in urban markets for pesticide residues because this is not being done by a food safety agency, or, as a thesis report put it, to analyse the content of powdered milk because ‘these milks do not seem to be under any kind of regular control’ (Kane 2001). Similarly, enquiring about what pharmacists know about managing intoxication makes sense because, at the time, they received no official advice or protocols, nor did they have access to a fully functioning poison control emergency helpline. If these networks were fully operational, in the way *thésards* and their supervisors imagine them to be elsewhere, particularly in France, then the work done by *thésards* would not count as original research but as routine regulatory or preventive health work. Instead, many theses conclude with recommendations for the implementation of legislation or the resourcing of government labs and agencies that would extend, in time and space, through monitoring, regulation and public information, what *thésards* were able to achieve on a small scale. For example, one thesis that tested artisanal peanut oils for contamination by aflatoxins, a carcinogenic fungus that grows on various foods (and is significant to the Senegalese economy in relation to its main export crop, peanuts) recommended ‘the establishment of a *real* food surveillance policy including *regular and systematic* controls’ (Dia 1997, emphasis mine).

Thus thesis work is not just research *about* a public health issue, such as exposure to toxic chemicals, food quality, or the quality of medicines, to name three frequent topics. It is also research that, in many cases, *would be* routine public health if it were inserted in official circuits of information about risk and safety. This is different from research *appearing as* public health, as is the case of experimental trials that provide healthcare not otherwise accessible, as well as from circumscribed health interventions that, by serving to evaluate delivery methods or outcomes, become experimental; yet, as in these other cases, there is a conflation of research with public health that arises from limitations in the temporal, spatial and social coverage of existing health prevention and care (Nguyen 2009; Lema *et al*. 2009).

The relationship of thesis work to routine prevention – its gaps and possible extensions – is important because this, I suggest, imparts to it a significance for *thésards* that exceeds its scientific value and concrete impact. During thesis work, *thésards* ‘sample’ the professional role of operating and extending the reach of government public health institutions, a role most do not think they will play again. Evoking this work as a ‘parenthesis’ comments on the contraction of career possibilities, but also on the uneven reach of public health from its institutional nodes to ‘street level’. In their modest contributions and ambitious (if usually unheeded) recommendations, theses project the ideal of fine-meshed and continuously active networks for the circulation of information about contamination and intoxication. Indeed, it is in relation to the absence of optimal networks of control (which have never existed in Senegal yet appear to remain relevant as realistic aspirations) that the spatial significance of thesis fieldwork as street-level work emerges. Even when *thésards* do *not* move across large chunks of the city, and though they do not seek to map the circulation of contamination or nonconformity, they put strategic locations – those which extend coverage to broader publics – ‘on the map’ of regulation and prevention.

## SPACES OF QUALITY

To illustrate this last point, let us take a closer look at how a subset of theses engages with a particular public health issue: drug quality. The main institution responsible for monitoring drug quality is the LNCM. Created on paper in 1979, the laboratory was not supplied with space, equipment and staff until 1987. Lab material was basic, allowing a limited range of tests. But the main problem was one of circulation. The LNCM's mandate was to respond to demands for testing by the directorate of pharmacy, the structure responsible for authorizing the entry of drugs into the Senegalese market and healthcare system, and for regulating their sale. Yet the demand, according to Mame Ndack, a senior technician who has worked in the lab since its inception, was ‘nil’.^10^

From 1998, the laboratory was relocated and extensively re-equipped, but the flow of drugs for testing increased unevenly. Substantial investment in the lab's facilities was mobilized by ‘political will’ emanating from a Minister of Health sensitized to the public health issue of fake, contraband and substandard drugs.^11^ But the lab also received support – and pressure – from foreign and international organizations concerned with the control of specific substances, such as the yellow fever vaccine produced by the Institut Pasteur of Dakar (and which the LNCM ‘releases’ for export), and especially the medicines used in specific public health programmes targeting malaria, tuberculosis and HIV/AIDS. For anti-malarials, which the US government funds through its President's Malaria Initiative, the US Pharmacopeia has launched a programme to sample and test drugs in five sentinel sites across the country (one of which is in the Dakar suburb of Guediawaye; the others are in Richard-Toll, Touba, Velingara and Kaolack) using a field-testing kit called the Minilab.^12^ For most of the 2000s, the fieldwork for the Minilab programme was overseen by the university's Laboratory of Analytical Chemistry and Toxicology. Substances that showed non-conformity were sent to the LNCM, which has received support through this programme, for confirmation. In addition to the national malaria control programme, other disease control programmes receive grants from the Global Fund to fight AIDS, Tuberculosis and Malaria (GFATM) that come with both requirements and earmarked funds for drug quality testing, allowing them to provide the LNCM with the reference substances needed to monitor specific drugs.^13^ Although field testing is still limited to the malaria programme, the HIV/AIDS programme has instituted storage site inspections.

Thus the programmes and their international funders, rather than the directorate of pharmacy, have increased the demand for drug testing and its reach across the national territory and ‘down’ to the level where medicines are stored, distributed and administered. This permits statements that imply mobile, fine-meshed and extensive drug-quality monitoring: according to Dr Sarr, who holds a joint appointment as the head of one of the LNCM's sections and as a junior staff member in the university laboratory, the system ‘enmeshes [*maille*] the whole Senegalese territory … it's a system that's really dynamic’, while Dr Ciss, the LNCM's recently retired former director, often declared that quality was tracked ‘down to the patient's bed’.^14^ Yet their own accounts, and those of other senior staff of the LNCM, make it clear that only a fraction of drugs are caught by this net. They readily admit that the directorate of pharmacy remains reluctant to send drugs for testing, despite the requirement that medicines applying for Senegalese approval be monitored, and its designation as the ‘political’ arm of drug-quality monitoring on a national scale. And despite the LNCM's cutting-edge equipment, mostly purchased by the Senegalese state (allocating from a budget made up of foreign funding), it remains difficult for staff to obtain expensive, single-use reference substances for determining the conformity of drugs other that those provided by the programmes financed by the Global Fund and USAID (among others).

‘So what about medicines outside the programmes?’ I asked Sarr. He answered: ‘We can control them through research, for example with students who do theses.’ Thesis research does not have to follow the networks traced by official regulatory and resource flows. *Thésards* can go downstream, to ‘street level’, to sample drugs that have been authorized for sale in Senegal without batch testing or field monitoring – exposing, according to Sarr, ‘the dysfunctional relation [of the LNCM] with the directorate of pharmacy. It's a Damocles sword above their heads.’ *Thésards* were recruited as fieldworkers when the University lab ran the USP Minilab programme, helping to sample and test malaria medicines in the sentinel sites. But they also extended the sampling to other categories of drugs such as antibiotics and pain relievers. *Thésards* can also sample and test medicines sold illicitly in informal markets, which the LNCM refuses to do as an official position.^15^ Even before 1998, when the lab was modestly equipped and the official demand for testing was ‘nil’, Ndack reports that *thésards* used the lab to test medicines, notably those sampled from the illicit market.^16^ Pharmacies, healthcare facilities, homes and especially the illicit market emerge as strategic, previously neglected locations for the extension of drug quality control. Sampling in these locations promises the protection of broader populations, including those who suffer from conditions not targeted for massive global health investments, or those not able to afford to pay for guaranteed quality in legal pharmacies.

Thesis work has a limited impact in terms of closing gaps in regulation: it is evoked by faculty members such as Sarr as a call for (rather than the enactment of) an extension of the spatial and vertical coverage of public health institutions in which they occupy or seek to create positions of responsibility. There are similar dynamics between the partial coverage of official monitoring and reliance on thesis work as its projected extension in the areas of food quality and poison control. In a 2008 feasibility study for setting up a Senegalese food security agency, food control capacity was described as lacking coordination and means, and as being oriented towards monitoring products entering the market rather than those circulating within it (which might be artisanal or contraband, and potentially contaminated by poor handling and storage) (République du Sénégal 2009). Much greater, mainly private, investments have been made to ensure that fish, vegetables and peanuts for export meet standards for heavy-metal, pesticide and aflatoxin levels in the markets for which they are destined. Thus, when *thésards* collect peanut products or vegetables in local markets and homes, they are marking out spaces that official sampling and testing does not but must reach, if it is to have any effect on the majority of local consumers. In this way, *thésards* contribute to the Faculty of Pharmacy's ambition to reinforce the position of the university lab as the central node of a tighter food security system.

The head of the toxicology section of the lab has also been the driving force and first director of the CAP. *Thésards’* surveys of intoxication rates in hospital statistics helped bolster the case for the CAP in the early 2000s. When I spent time in the CAP's still unfinished building in 2010, staff had only begun to collect and diffuse information about intoxication, notably through a survey of snakebites (partly supported by a major cotton-growing company and a firm that produces anti-venom therapy) and an emergency helpline. The director was looking for funding to do a survey of domestic chemical risks; caustic soda was known to be the cause of frequent acute intoxications in children. By 2013, a *thésard* was getting ready to do this survey. Often working without grants, using their modest bursaries for bus fare, *thésards* have been sent out on the streets of Dakar, across diverse neighbourhoods, to administer questionnaires on knowledge and risks of intoxication in pharmacies, homes, peri-urban fields and beauty salons. The students I interviewed said they not only asked questions but tried to inform and ‘sensitize’ their respondents to the risks posed by medicines and chemicals – although this was not part of their research objectives.

It is hard to say for sure how much *thésards* share their supervisors’ aspirations for the street-level extension of drug and food quality regulation and the circulation of information about exposure to toxic chemicals. Comments scribbled in margins of thesis reports kept in the lab's filing cabinets, and similarities across reports in the wording of justifications and recommendations, show that supervisors influence the images of potential regulation projected by *thésards*. Yet in undertaking thesis work they are temporarily caught up in these aspirations (something that does not happen in lectures, according to one student). The tasks of locating sites across the city, collecting samples and administering surveys, manipulating substances and data, and formulating recommendations to ‘the public’ and ‘the authorities’ allow them to enact very concretely the orientation of their position and qualifications as pharmacists towards public health goals. Few if any *thésards* in any given year can hope to join their supervisors: recruitments for lab positions, which also provide access to appointments in government health institutions such as the LNCM and CAP as well as the pharmacies of public teaching hospitals, are limited to a handful of graduates every few years (in the toxicology section, an assistant told me there had been recruitments in 2006 and 2012, and ‘who knows when the next will be’). Only during the thesis, then, can most take part in their supervisors’ ‘missions’ to conduct research and protect public health, perhaps also sharing their aspirations to extend the reach of food, drug and poison control to broader and more inclusive populations.

Indeed, it is mainly through *thésards’* modest unpaid work that such extended circuits of public health can be outlined. At the university, pharmacists lack time and equipment to do much research, while the government institutions they run have yet to reach ‘street level’ through inspection, monitoring, surveys or guidelines (nor have they in the past: sources suggest there was never more than one pharmacy inspector for Senegal).^17^ The political economies of global health and export commerce, and as yet unfinished projects of national control, make the current monitoring of poor quality and toxicity selective. Not only do *thésards* help supplement faculty members’ research record (the latter readily told me that *thésards’* unpaid work enabled them to publish, though this concerns only a tiny fraction of theses). Through their modest labours and ambitious recommendations, *thésards* project much broader urban and national publics for Senegalese public health. They open up the imagination of what Senegalese pharmacists’ scientific and public responsibilities might be; of the ways in which they might produce, collect and diffuse knowledge for public health; and of the public health projects with which they might be associated.

When young graduates speak of their thesis work as a ‘parenthesis’, they evoke a juxtaposition of past, possible and probable roles for pharmacists. These roles penetrate their sense of history and their aspirations for the future, and they are associated with specific tasks but also with an ethos. Perhaps what they yearn for most is not to re-enact thesis work over the course of their careers but to keep the Senegalese pharmacist's versatility open, to be able to choose between and even combine the values and work of business, public service, research and care as a broad basis for their livelihoods and professional identities.

## CONCLUSION

Studies of African cities, youth and new ‘figures of success’ have emphasized inventiveness, adaptability and individualization as responses to economic, social and political change. New strategies of survival, accumulation and political expression are thus associated with historical and generational rupture, and with the affirmation of an entrepreneurial ethos and consumerist values. Yet not all urban Africans, young or old, may have ‘finished grieving for the old economic order’, as Petit and Mutambwa point out in a study of Lubumbashi residents’ perspectives on informal economic activity (2005). They suggest that recent and widespread access to formal, salaried work in Lubumbashi has made its residents slower, and perhaps more reluctant, than residents of Kinshasa, for example, to embrace new values and strategies of ‘making do’. By studying only what urban Africans and youth seek to gain in the new interstices, fracture lines and connections of increasingly informalized, privatized and globalized economies, we risk missing expressions of attachment to forms of stability, regulation and citizenship that are associated with older economic and political orders, and a sense of loss.

The ambivalence to ‘take advantage’ of current opportunities to make money expressed by young pharmacists may be partial and circumstantial. In other situations, or a few years down the line, when they have finally acquired their own pharmacies or are accumulating sales commissions, they may instead appear to embrace the entrepreneurial ethos. Yet, as I suggest in this article, it is worth taking seriously the critique and compromise they voice through this ambivalence, and thus to open up and make sense of fissures in what might be too smooth an account of the maintenance of Senegalese pharmacists’ successful destinies.

Today's young pharmacists may not be yearning for a real, faraway past of civil service, nationalism and an expansive state. But they do seem to want to do more with their expertise than seems possible in the careers they see laid out before them. The thesis, which so recently, but temporarily, carried ‘something more’ – knowledge, service, the possibility of public health – can be seen as an opening onto both past expectations and unfulfilled aspirations. *Thésards* briefly explore and extend public health in the city, and their fieldwork practices temporarily materialize and spatialize (through locations of neglect) its public as national and inclusive. At the same time, the parenthesis of thesis work opens onto past and future career trajectories, and the publics that these promise to engage. Recent graduates’ expressions of something like nostalgia for thesis work evoke a broader refusal of the apparent disjuncture between the scientific basis of pharmacists’ training – and its potential civic functions – and current opportunities and modalities for the deployment of their expertise. It juxtaposes the geography of urban commercial transactions they feel destined to enter with alternative – older and novel, enacted and imagined – geographies of scientific and public responsibility. Their current city appears as folded onto its own ability to acquire, consume and generate profit as a market. In the city of their thesis work, evoked with regret and hope, appears another type of space that extends through banal practices of investigation and regulation towards an ill-defined, but more inclusive ‘population’ that inhabits the nation.

## Footnotes

1 The names of former and current thesis students interviewed for this project have been replaced by pseudonyms, given that the stated goal of the project was a collective rather than individual description of student trajectories. Most academic staff members, who were interviewed as part of a broader project on the history of pharmacy and toxicology, consented to the use of their real names.

2 The students were recruited for this study through their current or former supervisors at the Faculty of Pharmacy. The small number (about 3–5) of students supervised by each faculty member each year made it difficult to recruit a larger sample in the brief time of a pilot project. During my two visits, no student was conducting fieldwork; it was thus impossible to conduct direct observation. All but two interviews were conducted at the Faculty of Pharmacy. Although interviewees offered little indication that they regarded me as a potential source of opportunities (for example by bringing up bursaries or overseas studies), my position as a foreign academic and link to their supervisors, as well as my interest in their thesis work and professional ambitions, may have inspired such hopes.

3 Interview with Doudou Ba, Académie des sciences et techniques du Sénégal, 27 January 2009.

4 Souleymane Mboup was listed as one of 100 prominent Africans in the magazine *Les Afriques* in 2008, see <http://www.lesafriques.com/100-africains/souleymane-mboup-professeur-de-medecine.html?Itemid=195?articleid=9597>, dated 20 November 2008, accessed 20 November 2011. Balla Moussa Daffé's CV is on the Senegalese Academy of Science and Technology website, <http://www.asts.sn/asts/membre/cvasts/baladaffe>, dated 2004, accessed 2 November 2011. Mamadou Badiane is remembered for his regulatory work by Awa Marie Coll-Seck, then executive director of Roll Back Malaria (and later appointed as Senegal's Minister of Health), in her inaugural speech (Coll-Seck 2011).

5 Interview with Doudou Ba, February 2009; interview with Mounirou Ciss, February 2010.

6 Interviews with Babacar Niane, March and August 2010; Mamadou Fall, personal communication, August 2010 and February 2011.

7 Interview with Mounirou Ciss, February 2010. Interview with Amadou Moctar Dieye, February 2009.

8 Mamadou Fall, personal communication, February 2011; interview with Khalifa Sene, August 2011.

9 This is in part due to difficulties accessing lab equipment and supplies, as well as the limited scope of thesis-level work, but it is also related to broader shifts in methodology; see Tousignant (2013b).

10 Interview with Mame Ndack, Laboratoire National de Contrôle des Médicaments, Dakar, 18 January and 5 February 2013.

11 Interview with Mame Ndack.

12 Interview with Mounirou Ciss, Laboratoire National de Contrôle des Médicaments, Dakar, 26 January 2009; interview with Yerim M. Diop, Faculty de pharmacie, Université Cheikh Anta Diop, Dakar, 2 February 2010.

13 Interview with Mounirou Ciss, Laboratoire National de Contrôle des Médicaments, Dakar, 22 February 2010 ; interview with Mamadou Sarr, Faculty de pharmacie, Université Cheikh Anta Diop, Dakar, 18 January 2013.

14 Interview with Mamadou Sarr; interview with Mame Ndack.

15 On the high political stakes of the regulation of the illicit pharmaceutical market in Senegal, see Tousignant 2013a; Fassin 1986. The rationale for this policy at the LNCM was that the illicit market should be closed down, regardless of the quality of its medicines; thus demonstrating it to be good or bad would not change the recommended course of action.

16 Interview with Mame Ndack.

17 Drug quality and poison control, similarly, have never been fully operational at ‘street level’. Food inspection is a slightly different case, as it exceeds the control of conformity and toxic contamination in which pharmacists are involved. Meat and restaurant inspections, for example, have been more actively and densely covered.
